# Neutrophil–Lymphocyte–Platelet Ratio for Predicting Bacteremia in Immunosuppressed Cancer Patients: A Retrospective Diagnostic Accuracy Study

**DOI:** 10.3390/biomedicines14051170

**Published:** 2026-05-21

**Authors:** José Manuel Martinez, Ana Espírito Santo, Pedro Leite, Ana Pinho, Ana Rita Carneiro, Ana Maria Oliveira, Diana Ramada, Rui Medeiros

**Affiliations:** 1 Doctoral Programme in Biomedical Sciences, ICBAS School of Medicine & Biomedical Sciences, University of Porto, 4050-313 Porto, Portugal; diana.ramada@ipoporto.min-saude.pt; 2 Clinical Oncology Group, IPO Porto Research Center (CI-IPO Porto), 4200-072 Porto, Portugal; ana.esanto@ipoporto.min-saude.pt; 3 RISE—Associate Laboratory (Health Research Network), 4200-072 Porto, Portugalruimedei@ipoporto.min-saude.pt (R.M.); 4 Porto Comprehensive Cancer Centre Raquel Seruca (Porto.CCC), 4200-135 Porto, Portugal; 5 Portuguese Oncology Institute of Porto (IPO Porto), 4200-072 Porto, Portugal; ana.pinho@ipoporto.min-saude.pt (A.P.); ana.r.carneiro@ipoporto.min-saude.pt (A.R.C.); ana.lopes.oliveira@ipoporto.min-saude.pt (A.M.O.); 6 Epidemiology, Outcomes, Economics & Management in Oncology Group, IPO Porto Research Center (CI-IPO Porto), 4200-072 Porto, Portugal; 7 Oncology Nursing Research Unit, IPO Porto Research Center (CI-IPO Porto), 4200-072 Porto, Portugal; 8 Molecular Oncology and Viral Pathology Group, IPO Porto Research Center (CI-IPO Porto), 4200-072 Porto, Portugal

**Keywords:** bacteremia, sepsis, neutrophil–lymphocyte–platelet ratio (NLPR), inflammatory biomarkers, immunosuppressed cancer patients, blood cultures

## Abstract

**Background:** Early identification of bacteremia in immunosuppressed cancer patients remains difficult, especially in neutropenia. This study evaluated the diagnostic accuracy of NLR, PLR, and NLPR for identifying bacteremia and sepsis in patients undergoing blood culture episode. **Methods:** We conducted a retrospective diagnostic accuracy study at a tertiary oncology center between January 2023 and December 2024. All bacteremia identified were included as cases. Culture-negative episodes were subsequently sampled as controls using a frequency-matching strategy. Hematological parameters were obtained within ±24 h of first blood culture episode. Diagnostic performance was assessed using ROC curve analysis and multivariable logistic regression. **Results:** Of 369 screened episodes, 337 from 323 unique patients were included after excluding 31 records. NLPR showed the highest accuracy for bacteremia (AUC 0.730; 95% CI 0.671–0.788). The optimal cut-off was 0.038 (sensitivity 69.2%, specificity 72.3%) and remained consistent after excluding episodes with antibiotic therapy (AUC 0.768), corticosteroids (AUC 0.708), or growth factor use (AUC 0.718). In severe neutropenia, NLPR showed the highest accuracy (AUC 0.887; 95% CI 0.797–0.978). In multivariable analysis (n = 304), NLPR remained independently associated with bacteremia (*p* < 0.001), with good model discrimination (AUC 0.815; 95% CI 0.763–0.866). Diagnostic performance for sepsis was lower and not statistically significant. **Conclusions:** These findings suggest that NLPR may represent a simple, inexpensive, widely accessible adjunctive biomarker to support early bacteremia risk stratification in immunosuppressed cancer patients, particularly in patients with severe neutropenia. Although its overall discrimination was comparable to isolated lymphocyte count, NLPR may provide clinically relevant contextual information by integrating multiple dimensions of immune dysregulation. Further prospective multicenter validation is warranted.

## 1. Introduction

Some of the most serious complications in immunosuppressed patients, especially neutropenic, are bloodstream infections and sepsis. This frequently leads to adverse outcomes and the early identification of patients at risk is still one of the many challenges faced in routine practice [1]. In most clinical settings, blood cultures remain as the key element of microbiological confirmation of bacteremia and guide for antimicrobial therapy [2].

In the absence of definitive microbiological confirmation, clinicians are often forced to initiate empirical therapy [3,4]. The blood culture sensitivity in these patients may be compromised by prior antibiotic exposure, low circulating bacterial load, and pre-analytical variability, which can possibly increase the risk of false-negative results and delayed diagnosis [1,5].

The neutrophil-to-lymphocyte ratio (NLR) shows the proportion between innate immune activation and adaptive immune suppression, a critical feature of systematic inflammation and infection. Elevated NLR values have been associated with adverse outcomes, including sepsis and critical illness [6,7]. Also, the platelet-to-lymphocyte ratio (PLR) shows the interaction between inflammatory and coagulation pathways [8,9], which are closely linked in the pathophysiology of sepsis [10].

Another relevant biomarker, the neutrophil-lymphocyte-platelet ratio (NLPR) has been suggested as a composite index that may even further reflect inflammatory burden and immune dysregulation [11]. Although there have been studies that have explored this association in general or critically ill populations, their diagnostic performance in immunosuppressed cancer patients is still not well defined [12]. For this reason, this study aimed to evaluate the diagnostic accuracy of NLR, PLR, and NLPR for the early identification of bacteremia and sepsis in blood culture collections of immunosuppressed cancer patients, in order to support early risk stratification and clinical assessment in this high-risk population.

## 2. Materials and Methods

### 2.1. Study Design and Population

This retrospective diagnostic accuracy study was conducted, from January 2023 to December 2024, at the Portuguese Institute of Oncology (Porto), a 360-bed tertiary oncology center. This hospital-based sampling frame shows primarily healthcare-associated infections, in which patients with polymicrobial bacteremia, mycobacteremia, aged <18, were excluded from the study. The initial blood culture collection (T0) was obtained due to a clinical suspicion of infection related to an infectious event. The blood cultures that resulted in bacteremia were included as cases, whereas culture-negative results were sampled as controls, through a frequency-matching strategy. If all blood cultures were negative, only the first negative collection was considered for analysis. Taking into consideration the oncological population and the possibility of prolonged or repeated hospitalizations, the patients could contribute more than one infectious event, if those events were clinically distinct.

A case-enriched design was adopted to ensure adequate representation of bacteremia episodes. All eligible episodes with microbiologically confirmed bacteremia were included as cases. Culture-negative episodes were subsequently selected as controls using a 2:1 case–control sampling strategy, with frequency matching by sex and age (±5 years). When multiple potential controls met the matching criteria, control episodes were selected at random.

The following variables were extracted from electronic medical records: age, sex, underlying diagnosis and clinical conditions at the time of blood culture collection (included ongoing systemic antibiotic therapy, corticosteroid therapy, and use of hematopoietic growth factors). The hematological parameters were obtained from routine complete blood count analysis within a predefined time window of 24 h before to 24 h after blood culture collection, including absolute neutrophil count and derived inflammatory ratios: NLR, PLR, and NLPR.

All hematologic components used to compute the NLR, PLR, and NLPR were expressed in the same unit, cells per cubic millimeter (cells/mm^3^), ensuring consistency across all ratio calculations. The NLR is calculated by dividing the absolute neutrophil count by the absolute lymphocyte count, while the PLR is calculated by dividing the platelet count by the absolute lymphocyte count [6,7,9,11,12]. The NLPR was computed by dividing the neutrophil to lymphocyte ratio by the platelet count, which is mathematically equivalent to the absolute neutrophil count divided by the product of lymphocytes and platelets [11]. Severe neutropenia was defined as ANC < 0.5 × 10^9^/L (500 cells/mm^3^), corresponding to Grade 4 neutropenia according to CTCAE version 5.0 [13].

The primary outcome of this study was bacteremia and the secondary outcome was sepsis at day 1, defined according to Sepsis-3 criteria as suspected or confirmed infection associated with an acute increase of ≥2 points in the Sequential Organ Failure Assessment (SOFA) score. The SOFA scores were calculated using clinical and laboratory data available within 24 h of blood culture collection.

### 2.2. Statistical Analysis

An a priori power analysis for diagnostic accuracy (ROC curve) was conducted using the pROC package, assuming an expected AUC of 0.80, α = 0.05, power = 0.80, and a case–control ratio of 2:1 (κ = 2), resulting in a minimum requirement of approximately 30 total observations, which the final analytic sample exceeded.

The variables were summarized according to its type: continuous variables used medians and interquartile ranges (IQR), whereas categorical variables used counts and percentages. The comparisons between groups focused on biomarker distributions according to bacteremia status, through appropriate nonparametric or categorical tests.

The diagnostic performance of NLR, PLR, and NLPR for bacteremia and sepsis was evaluated using ROC curve analysis, with calculation of the AUC and corresponding 95% confidence intervals. Furthermore, the pairwise comparisons of AUCs utilized the DeLong test, while the optimal cut-off values were identified through the Youden index.

Multivariable binary logistic regression models were constructed for bacteremia, the primary outcome. Variables considered clinically relevant or potentially associated with infection risk (i.e., age, sex, underlying diagnosis, systemic antibiotic therapy, corticosteroid therapy, and use of hematopoietic growth factors) were included in the adjusted model and adjusted odds ratios (aORs) with 95% confidence intervals were reported. Given the limited discriminative performance in unadjusted analyses of sepsis as a secondary outcome and its adjusted models were not pursued.

All analyses were performed using R statistical 2022 Posit Software (R Foundation for Statistical Computing, Vienna, Austria) and data available for each variable. A two-sided *p*-value < 0.05 was considered statistically significant.

### 2.3. Ethical Considerations

The study protocol was reviewed and approved by the institutional ethics committee of IPO Porto (ethics approval CES.072_25). The study was conducted in accordance with the Declaration of Helsinki and applicable national regulations.

## 3. Results

### 3.1. Study Population

A total of 369 blood culture episodes were screened, of which 337 eligible episodes (323 unique patients) were included in the analysis. Repeated episodes occurred in 30 patients (9.3%); given their low frequency and separation by clinically distinct events, no adjustment for within-patient correlation was performed. The median age was 64.1 years (IQR 56.0–74.0), and 60.4% were male. Severe neutropenia was present in 15.1% of episodes. Baseline characteristics according to bacteremia status are summarized in Table 1.

### 3.2. Diagnostic Performance of Inflammatory Ratios

**Figure 1:**
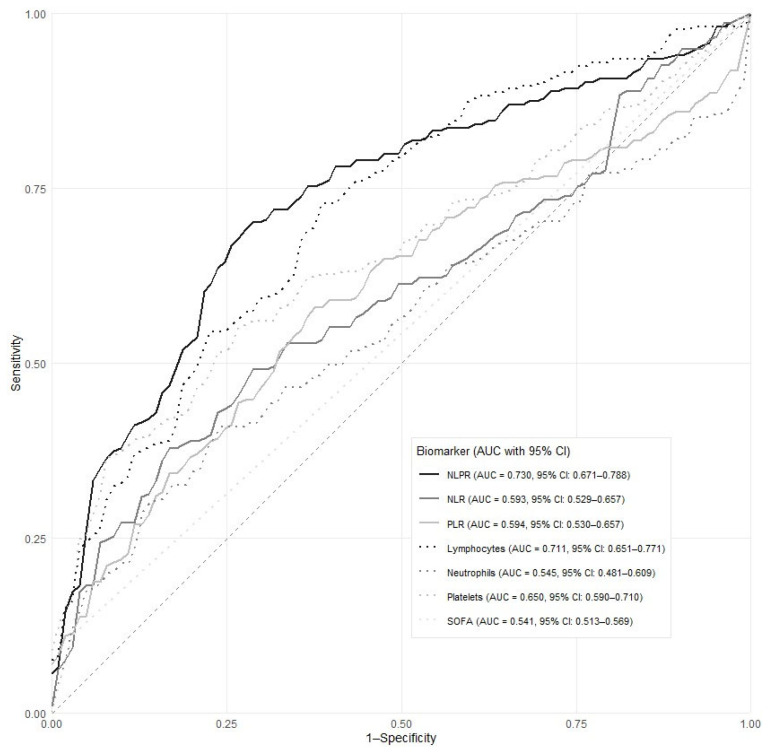
Receiver operating characteristic curves for predicting bacteremia.

NLPR values were significantly higher in bacteremic compared with culture-negative episodes (median 0.06 [IQR 0.03–0.15] vs. 0.02 [IQR 0.01–0.04]) (Table 1), whereas differences for NLR and PLR were less pronounced. NLPR demonstrated the highest diagnostic accuracy for bacteremia among the evaluated biomarkers (AUC 0.730; 95% CI 0.671–0.788), significantly outperforming both NLR (AUC 0.593; 95% CI 0.529–0.657) and PLR (AUC 0.594; 95% CI 0.530–0.657). In contrast, isolated neutrophil count and SOFA showed limited discriminatory ability, with AUCs of 0.545, 95% CI 0.481–0.609 and 0.541, 95% CI 0.513–0.569 respectively. Comparative ROC analyses showed that NLPR significantly outperformed isolated neutrophil count (*p* = 0.0001), SOFA (*p* < 0.001), NLR (*p* < 0.001), and PLR (*p* = 0.002) for bacteremia discrimination. Isolated lymphocyte count showed acceptable discriminatory ability for bacteremia, with an AUC of 0.711 (95% CI 0.651–0.771), outperforming isolated neutrophil count, SOFA score, NLR, and PLR. Although NLPR demonstrated numerically higher discrimination, no statistically significant difference was observed between NLPR and isolated lymphocyte count (*p* = 0.800) (Figure 1). Exploratory analyses of biomarker variability showed greater relative dispersion for lymphocyte counts (coefficient of variation [CV] = 2.5) compared with neutrophils (CV = 0.93) and platelets (CV = 0.78), suggesting that lymphocyte depletion may represent an important contributor to the discriminatory signal observed for NLPR in this cohort.

The optimal NLPR cut-off was 0.038, yielding a sensitivity of 69.2% and specificity of 72.3%, corresponding to a positive likelihood ratio of 2.50 and a negative likelihood ratio of 0.43. For sepsis at day 1, NLPR showed higher discrimination (AUC 0.558; 95% CI 0.491–0.625) compared with NLR and PLR; however, the overall performance was limited, and adjusted analyses were not pursued for this secondary endpoint.

### 3.3. Subgroup and Sensitivity Analyses

The diagnostic performance of NLPR for bacteremia showed no significant differences with systemic antibiotic therapy and corticosteroid use. In these stratified analyses, the AUC was 0.768 (95% CI 0.69–0.84) in episodes without antibiotic exposure and 0.652 (95% CI 0.57–0.73) in antibiotic-treated episodes (*p* = 0.139). Similar results were observed according to corticosteroid use, with AUCs of 0.708 in non-corticosteroid episodes and 0.731 in corticosteroid-treated episodes (*p* = 0.794). Thus, the overall discrimination remains similar to the one observed in the full cohort, where the AUC was 0.730 (95% CI 0.671–0.788). Subgroup-specific estimates were not interpreted further, as they were considered statistically unstable due to the reduced number of growth factor-treated episodes (*n* = 13). Additional exploratory analyses that compared hematologic biomarkers according to microbiological classification of bloodstream infections, including Gram-negative, Gram-positive, and fungal episodes, were performed (Supplementary Table S1). Exploratory analyses according to microbiological etiology showed similar hematologic patterns across fungal, Gram-negative, and Gram-positive bloodstream infections. Gram-negative episodes demonstrated the highest NLPR values (0.07 [IQR 0.03–0.19]), followed by fungal and Gram-positive infections (both 0.06), whereas culture-negative episodes showed substantially lower values (0.02 [IQR 0.01–0.04]) (*p* < 0.001). In contrast, isolated neutrophil counts did not significantly differ between microbiological groups (*p* = 0.400).

When stratified by severe neutropenia at the time of blood culture collection, NLPR values were significantly higher in bacteremic episodes (0.07 [IQR 0.03–0.12]) compared to non-bacteremic episodes (0.01 [IQR 0.00–0.01]) (*p* < 0.001). The diagnostic performance of NLPR also differed substantially according to the patient’s neutropenia status. In patients without severe neutropenia (*n* = 263), NLPR showed acceptable discrimination for bacteremia, with an AUC of 0.700 (95% CI 0.635–0.765), a value similar to the full cohort. In contrast, in patients with severe neutropenia (*n* = 51), NLPR demonstrated a showed numerically higher diagnostic accuracy in this exploratory subgroup, with an AUC of 0.887 (95% CI 0.797–0.978), and a statistically significant difference between groups (*p* = 0.001). In comparative analyses within the severe neutropenia subgroup, NLPR showed numerically higher discrimination than isolated lymphocyte count AUC of 0.806 (95% CI 0.663–0.948), although no statistically significant difference was observed (*p* = 0.520). The performance of NLPR biomarker in severe neutropenia also stays consistent in sensitivity analyses restricted to patients not receiving concurrent therapies. However, these findings should be interpreted cautiously due to the limited sample size of this exploratory subgroup.

### 3.4. Multivariable Analysis

In order to account for potential confounding factors, a multivariable logistic regression model of complete cases (*n* = 304) was developed including NLPR, age, sex, underlying diagnosis, current antibiotic therapy, corticosteroid therapy, and growth factor use (*n* = 304 episodes). This analysis showed that NLPR remained independently associated with bacteremia, with an aOR of 1.12 per 0.01 increase (95% CI 1.07–1.19; *p* < 0.001). In the same manner, current antibiotic therapy (aOR 6.26, 95% CI 3.01–14.4; *p* < 0.001) and corticosteroid therapy (aOR 2.96, 95% CI 1.37–6.93; *p* = 0.008) were also independently associated with bacteremia. In opposition, the use of growth factors did not reach statistical significance and age, sex, and diagnosis were not independently associated with the outcome. The adjusted model demonstrated good discriminative ability, with an AUC of 0.815 (95% CI 0.763–0.866). No evidence of multicollinearity (all VIFs ≤ 1.16) was observed (Table 2).

Additional analyses explored whether the association between NLPR and bacteremia differed according to the underlying oncological diagnosis. In multivariable models including an interaction term between NLPR and diagnosis category, no statistically significant interaction was observed (*p* = 0.112), indicating consistent discriminative performance across patients with hematologic malignancies (AUC 0.681, 95% CI 0.55–0.81) and solid tumors (AUC 0.747, 95% CI 0.68–0.81).

## 4. Discussion

### 4.1. Clinical Relevance and Primary Findings

The main finding of this study showed that NLPR had a superior diagnostic performance when compared to NLR and PLR. This finding suggests that this biomarker may be a more reliable sign of infection-related risk in this vulnerable population. It also demonstrated a substantially higher accuracy in patients with severe neutropenia, a rather surprising finding, as conventional inflammatory biomarkers in this subgroup often perform poorly due to profound treatment-related cytopenias [2,3,6]. This finding suggests that NLPR may retain discriminatory performance even in the setting of severe immunosuppression, where early identification of bacteremia is particularly challenging yet critically important [1,2,3,6]. In this case, sepsis represents established organ dysfunction that may develop later and be impacted by a number of non-infectious factors in cancer patients, while bacteremia represents a clinically proximal and objective endpoint at the time of blood culture collection. The reliability of diagnostic analyses based solely on sepsis is further limited by the small number of isolated sepsis cases and the partial overlap with bacteremia. Therefore, the focus on bacteremia as the main outcome in this study is supported by the fact that the biomarkers investigated are more likely to be informative as a predictor of the presence of bloodstream infection than diagnosing organ dysfunction. NLPR’s strength as a biomarker in complex oncological populations is further supported by its consistent performance across sensitivity analyses that do not include concurrent therapies [11,14,15]. The markedly lower performance of isolated neutrophil count compared with NLPR reinforces the concept that bacteremia-related immune dysregulation in immunosuppressed cancer patients is multidimensional rather than dependent on a single hematologic component. Similarly, the poor discrimination observed for SOFA suggests that hematologic alterations may precede overt organ dysfunction in this population. NLPR reported the highest overall AUC among the evaluated composite biomarkers and significantly outperformed isolated neutrophil count, SOFA, NLR, and PLR. However, its performance was comparable to isolated lymphocyte count, suggesting that lymphocyte depletion may represent an important driver of the observed discriminatory signal in this population. This finding suggests that lymphocyte depletion may play a central role in the host response associated with bacteremia, potentially reflecting impaired adaptive immune competence rather than classical neutrophil-driven inflammatory activation. The comparable performance between NLPR and isolated lymphocyte count observed in this cohort may reflect the profound immune dysregulation characteristic of immunosuppressed cancer patients, in whom lymphocyte depletion appears to represent a dominant biological signal. The substantially greater relative variability observed for lymphocyte counts compared with neutrophils and platelets may partially explain the comparable performance between isolated lymphocyte count and NLPR observed in this cohort. Given that lymphocyte count is incorporated as the denominator component of the NLPR formula, fluctuations in lymphocyte values may have a more substantial influence on the resulting biomarker values, particularly in profoundly immunosuppressed populations, as supported by our data showing that lymphocyte levels are the key driver of NLPR. This may also explain why the relative contribution of composite biomarkers such as NLPR differs across clinical settings, as the balance between neutrophil activation, lymphocyte suppression, and platelet consumption is likely context-dependent.

Furthermore, even after controlling demographic characteristics, underlying diagnoses, and concurrent treatments, the multivariable analysis carried out verified that NLPR is independently linked to bacteremia. This result suggests that NLPR’s predictive value is independent of clinical context or treatment-related variables and represents a strong biological indicator of infection risk in immunocompromised cancer patients. Furthermore, the use of corticosteroids and current antibiotic therapy were also independently linked to bacteremia; together, these variables may capture complementary dimensions of infection risk, including host inflammatory response, clinical severity, and treatment-related immunosuppression. The adjusted model demonstrated improved discriminative ability, when compared with individual biomarkers, supporting that the inclusion of NLPR with key clinical variables may enhance early risk stratification at the time of blood culture collection. The absence of a significant interaction between NLPR and underlying diagnosis suggests that its association with bacteremia is relatively stable across different oncological populations.

The adjusted model showed that severe neutropenia did not provide additional independent information. This is likely due to the fact that NLPR incorporates neutrophil counts as part of its calculation and may capture the impact of neutrophil depletion, in combination with other hematological components. Therefore, NLPR may represent a broader marker of the immune status than absolute neutrophil count alone. From a clinical perspective, this indicates that NLPR may serve as a surrogate marker for complex hematological alterations, including neutropenia, without requiring separate consideration of each component.

### 4.2. Biological Rationale and Comparative Performance of NLPR

As discussed previously, NLPR combines neutrophil, lymphocyte, and platelet information into a single index, whereas NLR reflects the balance between innate immune activation and adaptive immune suppression and PLR integrates inflammatory and coagulation pathways. Therefore, the observed advantage of NLPR is biologically plausible. This integrated approach may better capture the complex immune dysregulation of immunosuppressed cancer patients, who commonly have baseline cytopenias and treatment-related immune alterations, and may result in a more stable and discriminative marker [1,11]. Although isolated lymphocyte count demonstrated comparable overall discrimination to NLPR, composite hematologic indices may still provide clinically relevant contextual information by integrating multiple dimensions of immune and inflammatory dysregulation. In immunosuppressed oncological populations, where treatment-related cytopenias frequently coexist, composite biomarkers may better reflect the complexity of the host response than isolated cell counts alone. This may partially explain the numerically higher discrimination observed for NLPR in severe neutropenia. These findings also raise the hypothesis that the biological contribution of each hematologic component within composite inflammatory indices may be context-dependent and influenced by the underlying immune status of the studied population.

Although NLPR showed superior diagnostic performance when analyzed as an isolated biomarker, not a single laboratory parameter can fully capture the infection risk in this population [12,14,15]. Among recent international studies on baseline biomarkers, the role of NLPR and related ratios in cancer and sepsis prediction has been reinforced [16,17,18]. In previous studies, selected biomarkers have also been reported as having a higher diagnostic performance in other clinical settings. For example, procalcitonin has demonstrated a strong performance in emergency and pediatric populations, while its discriminative ability is more limited in hematological diseases. In emergency settings, procalcitonin (PCT) consistently demonstrates the strongest individual performance for predicting bacteremia, with an AUC of 0.835 (95% CI, 0.79–0.87; *p* < 0.001) [19]. In pediatric populations, PCT also showed superior diagnostic efficiency, reaching an AUC of 0.862 (95% CI: 0.819–0.906) [20,21]. More recently, machine learning models trained on hematological and clinical parameters have demonstrated the highest predictive accuracy, with AUCs up to 0.844 in external reports and 0.802–0.806 when applied to complete blood count/differential data in a large cohort [22]. Despite these promising results, the complexity and resource requirements of these approaches currently limit their routine implementation in clinical practice.

### 4.3. Clinical Implications and Integrated Risk Assessment

From a clinical perspective, a biomarker that supports early risk stratification at the time of blood culture collection is especially valuable [1,4]. In hemodialysis populations, recent evidence shows that NLR and CRP-to-albumin ratio also demonstrate a high predictive value for catheter-related bloodstream infections, achieving AUCs above 0.80 [23]. However, extrapolating these findings to immunosuppressed oncological populations must be cautious, as interpreting the C-reactive protein (CRP) may be limited due to lack of specificity and frequent baseline elevation, as a result of tumor-related inflammation, treatment-related tissue injury, or chronic inflammatory states. Therefore, in this context, CRP may only reflect inflammatory activity without discriminating between infectious and non-infectious causes [14]. In contrast, serum albumin represents a negative acute-phase reactant and marker of systemic inflammatory burden and physiological reserve, as it integrates several indicators, such as nutritional status, endothelial permeability, and hepatic synthetic function. Consequently, albumin may capture dimensions of host vulnerability that are not fully captured by inflammatory ratios alone [14,24].

This conceptual distinction between inflammatory burden and physiological reserve suggests that integrating complementary biomarkers may enhance multidimensional risk stratification, as opposed to isolated inflammatory markers. As previously discussed, risk stratification at the time of suspected infection is particularly challenging in immunosuppressed oncological populations, as clinical presentation may be elusive and laboratory parameters frequently influenced by treatment-related cytopenias [1]. Unlike other indicators, such as SOFA and MASCC risk index score, which require established organ dysfunction and capture baseline clinical risk, respectively, NLPR may be a routinely available biological sign when there is diagnostic uncertainty [2,3,4,25]. In this context, integrating simple hematological indices with markers of physiological reserve may introduce an additional pragmatic tool for clinical assessment and existing risk scores, in order to support early clinical assessment and risk stratification without adding another diagnostic burden [2,3,5,14]. As NLPR is derived from laboratory parameters that are readily available, it may represent a pragmatic adjunctive tool for early triage and clinical assessment, especially where rapid access to advanced biomarkers is limited.

### 4.4. Scope and Limitations

The focus on a well-defined immunosuppressed oncological population and the assessment of biomarkers at the time of collection improve the findings’ relevance and applicability. Additionally, the ROC curve analysis made it possible to compare each biomarker’s diagnostic performance objectively and avoid making assumptions about linearity or predetermined cut-off values. The use of a representative patient population further strengthens the validity and applicability of the results. Although the retrospective, single-center design without external validation may limit generalizability, the inclusion of interaction analyses represents a methodological strength, demonstrating the stability and consistency of the model across relevant subgroups.

Due to the fact that the analytic sample used a case-enriched design (all bacteremia cases plus randomly sampled culture-negative controls), the case–control ratio does not reflect underlying prevalence and the predictive values were not considered reliable indicators of clinical performance. Therefore, the emphasis was placed on prevalence-independent measures such as the AUC and likelihood ratios, complemented by sensitivity and specificity analyses. Additionally, the case-enriched design may not fully reflect the spectrum of patients encountered in routine clinical practice, potentially limiting external generalizability. These findings are promising but require external prospective validation. Furthermore, despite NLPR having a better diagnostic performance, it should be used as an additional tool to support early clinical assessment rather than replacing clinical judgment or microbiological testing. Further prospective validation in consecutive and multicenter cohorts is warranted.

## 5. Conclusions

These findings suggest that NLPR may represent a simple, inexpensive, widely accessible adjunctive biomarker to support early bacteremia risk stratification in immunosuppressed cancer patients, particularly in patients with severe neutropenia. Although its overall discrimination was comparable to isolated lymphocyte count, NLPR may provide clinically relevant contextual information by integrating multiple dimensions of immune dysregulation. Further prospective multicenter validation is warranted.

## Figures and Tables

**Table 1:** Baseline demographic, clinical, and laboratory characteristics according to bacteremia status.

Variable	Negative (*n* = 105)	Positive (*n* = 232)	*p*-Value ^1^
**Demographic variables**			
Age, years, median (IQR)	67.00 (55.50–74.00)	66.00 (57.00–75.00)	0.5
Male sex, *n* (%)	63 (60)	141 (60.8)	0.9
**Clinical characteristics**			
Hematologic malignancy, *n* (%)	22 (21.0)	60 (25.9)	0.3
Current antibiotic therapy, *n* (%)	16 (15.4)	91 (39.1)	<0.001
Corticosteroid therapy, *n* (%)	15 (14.3)	31 (35.8)	<0.001
Growth factor use, *n* (%)	6 (5.7)	7 (3.0)	0.2
**Hematological Parameters**			
Neutrophils, median (IQR)	5.97 (3.91–10.12)	5.08 (1.34–10.85)	0.2
Lymphocytes, median (IQR)	1.07 (0.58–1.65)	0.52 (0.16–0.85)	<0.001
Platelets, median (IQR)	216.00 (152.0–313.0)	144.00 (38.0–265.0)	<0.001
**Inflammatory ratios**			
NLR, median (IQR)	5.62 (2.44–9.93)	7.55 (2.27–19.15)	0.008
PLR, median (IQR)	216.7 (139.6–378.4)	311.1 (166.7–590.4)	0.007
NLPR, median (IQR)	0.02 (0.01–0.04)	0.06 (0.03–0.15)	<0.001
**Sepsis**			
Yes, *n* (%)	44 (42.7%)	172 (75.1%)	<0.001
**SOFA**			
High Risk, *n* (%)	3 (3.1%)	24 (11.2%)	0.037
**Mortality**			
Yes, *n* (%)	11 (10.7%)	43 (18.5%)	0.071

^1^ Wilcoxon rank sum test; Pearson’s Chi-squared test; Fisher’s exact test; Missing values < 6% for all biomarkers.

**Table 2:** Multivariable logistic regression analysis for bacteremia.

Variable	aOR (95% CI)	*p*
NLPR (per 0.01 increase)	1.12 (1.07–1.19)	<0.001
Age (years)	1.00 (0.98–1.03)	0.794
Sex	1.06 (0.59–1.91)	0.841
Diagnosis (hematologic vs. solid malignancy)	0.84 (0.40–1.71)	0.628
Current antibiotic therapy	6.26 (3.01–14.45)	<0.001
Use of growth factors	0.32 (0.07–1.36)	0.120
Corticosteroid therapy	2.96 (1.37–6.93)	0.008

## Data Availability

The original contributions presented in this study are included in the article. Further inquiries can be directed to the corresponding author.

## Supplementary Materials

The following supporting information can be downloaded at https://www.mdpi.com/article/10.3390/biomedicines14051170/s1, Supplementary Table S1: Baseline characteristics and biomarker distribution across microbiological groups.
