# Supplementary material for: Neutrophil–Lymphocyte–Platelet Ratio for Predicting Bacteremia in Immunosuppressed Cancer Patients: A Retrospective Diagnostic Accuracy Study
Source: Biomedicines. 2026 May 21;14(5):1170. doi: 10.3390/biomedicines14051170 (PMC13204695; doi:10.3390/biomedicines14051170)
Supplement: Supplementary file 1 [file biomedicines-14-01170-s001.zip › biomedicines-4276626-supplementary.pdf]

Table S1. Baseline characteristics and biomarker distribution across microbiological groups

Variable	Fungal BSI (n=8)	Gram-negative BSI (n=118)	Gram-positive BSI (n=106)	Culture-negative episodes (n=105)	p-value1
Lymphocyte count, median (IQR)	0.57 (0.40–0.66)	0.49 (0.13–0.79)	0.54 (0.24–1.05)	1.07 (0.58–1.65)	<0.001
Absolute Neutrophil count, median (IQR)	5.19 (3.25–6.93)	4.74 (1.30–9.29)	5.61 (1.39–12.03)	5.97 (3.91–10.12)	0.400
Platelets, median (IQR)	131.0 (59.0–200.0)	137.5 (30.0–239.5)	174.5 (60.0–303.0)	216.0 (152.0–313.0)	<0.001
NLR, median (IQR)	9.65 (3.96–12.60)	8.88 (2.24–21.62)	7.11 (2.20–16.31)	5.62 (2.44–9.93)	0.061
PLR, median (IQR)	326.79 (123.21–363.64)	290.00 (155.36–525.86)	325.02 (171.91–662.50)	216.67 (139.55–378.38)	0.036
NLPR, median (IQR)	0.06 (0.03–0.12)	0.07 (0.03–0.19)	0.06 (0.02–0.13)	0.02 (0.01–0.04)	<0.001
Sepsis, n (%)	4 (50.0)	91 (77.8)	77 (74.0)	44 (42.7)	<0.001

[1] Wilcoxon rank sum test; Pearson's Chi-squared test; Fisher's exact test.
